# The Utility of Indocyanine Green Near-Infrared Fluoroangiographyin Assessing Mastectomy Skin Flap Perfusion

**DOI:** 10.3390/jcm13237270

**Published:** 2024-11-29

**Authors:** Gian Paolo Azzena, Tito Brambullo, Federico Ricci, Laura Pandis, Alberto Marchet, Vincenzo Vindigni, Franco Bassetto

**Affiliations:** 1 Plastic and Reconstructive Surgery, Neuroscience Department, University of Padua, Via Giustiniani 2, 35128 Padova, Italy; gianpaolo.azzena@gmail.com (G.P.A.); tito.brambullo@unipd.it (T.B.); federico.ricci@aopd.veneto.it (F.R.); vincenzo.vindigni@unipd.it (V.V.); franco.bassetto@unipd.it (F.B.); 2 Division of Breast Surgery, Veneto Institute of Oncology IOV-IRCCS, Via Gattamelata 64, 35128 Padova, Italy; alberto.marchet@iov.veneto.it

**Keywords:** angiography, breast cancer, breast reconstruction, implant, mastectomy, skin flap

## Abstract

**Background:** Breast reconstruction with implants is now the preferred procedure following mastectomies. For successful reconstruction, accurate evaluation of the patient and skin flap viability is essential. This study aimed to analyze the impact of risk factors on mastectomy skin flap necrosis (MSFN) and the effectiveness of indocyanine green angiography (ICGA) in preventing complications. **Methods:** Fifty consecutive patients undergoing mastectomy were divided into two groups (arms A and B) based on the method of skin flap evaluation (ICGA vs. clinical assessment, respectively). Demographic details and the risk factor incidence were collected, and complication rates were compared between the two groups. Univariate analysis was conducted to identify correlations between mastectomy skin flap necrosis and the aforementioned risk factors. **Results:** The two groups showed comparable demographics and incidences of risk factors. Patients in arm A exhibited a lower rate of complications and reinterventions, although no significant differences were observed. Statistical analysis revealed a significant association between BMI, implant volume, and MSFN. **Conclusions:** ICGA proves to be an effective diagnostic tool for assessing skin flap viability. When coupled with meticulous patient selection, it aids in preventing complications.

## 1. Introduction

Breast cancer remains a formidable global health challenge, exerting a profound impact on women’s health and psychological well-being. Despite significant advancements in early detection and treatment modalities, breast cancer continues to represent the most prevalent malignancy affecting women, with substantial morbidity and mortality rates [1,2]. While mastectomy plays a crucial role in eradicating cancerous tissue and reducing the risk of disease recurrence, prosthetic reconstruction emerges as the predominant procedure (79.7% of cases) [1,2]. Owing to its reduced complication rates, shorter surgical durations, and absence of donor site morbidity, is often preferred over autologous reconstruction [3,4,5,6]. Over the course of medical advancements, a diverse array of surgical techniques has been explored and refined to enhance breast reconstruction outcomes. Among these, the subcutaneous and submuscular approaches, along with their respective variations, have received considerable attention. Historically, the subcutaneous approach to implant placement was associated with higher complication rates, including implant visibility, cutaneous rippling, and capsular contracture, leading to its initial dismissal in favor of submuscular techniques [7,8,9,10]. However, advancements in mastectomy techniques, preservation strategies, and implant materials have prompted a reevaluation of the subcutaneous approach, particularly in the context of prepectoral reconstructions [7,11,12]. The choice between immediate and delayed breast reconstruction represents another critical decision point for patients and clinicians. Immediate reconstruction offers the advantage of achieving breast reconstruction at the same time asmastectomy, minimizing the psychological impact of the breast removal and of a second intervention. On the other hand, immediate reconstruction is associated with a higher incidence of complications in comparison with the two-step method [13]. In particular, mastectomy skin flap necrosis (MSFN), which is considered the most critical complication in immediate reconstruction, has been reported to occur with rates up to 40% [14,15,16,17,18]. These complications pose significant challenges during the reconstruction pathway because they necessitate aggressive interventions, including multiple surgical revisions and potential implant removal, which can impact the timing of adjuvant therapy and exert psychological stress on patients. Identification of risk factors contributing to mastectomy skin flap necrosis occurrence is mandatory. Both surgical and patient-related factors have a role in this complication. Patient-related risk factors for MSFN encompass a range of factors, including smoking, advanced age, prior radiotherapy, a body mass index (BMI) over 30, diabetes, and a larger breast volume [19]. Giventhe significant impact of MSFN, there has been a concerted effort to develop vascular skin assessment tools to facilitate intraoperative decision-making for immediate implant reconstruction. While various methods have been proposed, clinical evaluation remains the cornerstone, incorporating assessments such as capillary refill, dermal bleeding, color, and skin temperature [20,21,22]. However, alternative methods, including Doppler echography and laser-color Doppler ultrasounds, have been explored, with particular attention being drawn to laser-assisted indocyanine green (ICG) angiography [22]. ICG angiography, initially employed in cardiac surgery, has emerged as a promising tool for evaluating tissue perfusion. By binding to plasma proteins and remaining intravascularly concentrated, ICG enables real-time evaluation of tissue perfusion upon excitation by infrared light [20]. Its favorable safety profile and short plasma half-life allow for repeated injections during surgery, facilitating a precise assessment of flap viability. Consequently, ICG angiography is regarded as the most effective means of overcoming operator-dependent limitations and ensuring flap viability with high sensitivity and specificity [23,24,25,26,27,28]. In light of these considerations, this study soughtto comprehensively analyze the risk factors associated with MSFN in mastectomy patients undergoing immediate implant reconstruction. Furthermore, it aimed to evaluate the impact of ICGA on flap viability assessment, particularly concerning complication rates, revision frequency, procedural duration, and hospital stay, in order to enhance patient outcomes and inform clinical practice.

## 2. Materials and Methods

### 2.1. Study Population

Ethical approval was obtained for this study from the CET-ACEV committee on 25 July 2024 with identification code: 510n/AO/24. This retrospective study included 50 patients who underwent immediate reconstruction. Inclusion criteria were patients up to 75 years old with breast cancer eligible for mastectomy and candidates for reconstruction with a breast implant, whether it be an expander or prosthesis. Patients eligible for autologous reconstruction and those over 75 years old who, due to comorbidities or surgical risk, were not candidates for reconstruction, were excluded from this study, since they did not receive any reconstruction. All the surgeries were performed by the same surgical team at the University Hospital of Padua from February 2020 to May 2022. The team included the same senior plastic surgeon and senior breast surgeon in both groups. The breast surgeon performed the mastectomies, while the plastic surgeon performed the reconstructions and scanning through the use of angiography. Age, body mass index, smoking, diabetes, breast size, mastectomy volumes, neoadjuvant therapies, history of breast surgery, and mastectomy technique were used to stratify the population. Throughout this study, patients were allocated to either arm A or B based on whether they underwent surgery at a facility equipped with angiography, thus giving them a random allocation. Upon completion of the mastectomy, the viability of the skin flap was assessed using laser-assisted indocyanine green angiography in arm A. Patients in arm B were assessed by clinical evaluation through examination of capillary refill, dermal edges’ bleeding, color, and skin temperature. All complications occurring within 60 days of the surgery were registered. Complications included MSFN, hematoma, seroma, wound dehiscence, and implant exposure. Complications were classified into major and minor based on the necessity of revision surgery. All subjects gave their informed consent for inclusion before participating in this study, which was conducted in accordance with the Declaration of Helsinki.

### 2.2. Surgical Technique

Different patterns of mastectomies were performed depending on tumor size and location and patient morphology. Nipple-sparing mastectomy (NSM) was performed only when the distance between the tumor and nipple areola complex (NAC) was at least 2 cm, ensuring oncological safety while preserving nipple sensation and aesthetics. To confirm the absence of subclinical nipple involvement, a retromammary specimen was routinely sent for intraoperative frozen-section biopsies. Patients with retromammary/nipple involvement on preoperative imaging or after intraoperative biopsy went through a skin-sparing mastectomy (SSM). Finally, a skin-reducing mastectomy (SRM) was performed on patients with large ptotic breasts. Whenever possible, a lateral incision in the shape an italic S was preferred in order to reduce the incidence of nipple necrosis [29]. In cases where NAC removal was necessary, an incision including the central quadrant was made. During the dissection, particular attention was paid to preserving the subcutaneous layer. The dissection was made by electrocautery without using epinephrine tumescence solution to avoid any possible intraoperative factors that could affect the ability of ICG angiography to evaluate skin perfusion. The mastectomy types are represented similarly for the two groups. After skin evaluation using an implant or expander sizer in both groups, and whenfinding a lack of skin perfusion (alteration in capillary refill, dermal bleeding, color, and skin temperature), the surgeon proceeded with the maneuvers he deemed most appropriate. In addition to trimming non-vital tissue, sometimes, it was necessary to choose a smaller implant than the one tested. In cases where the decrease in volume was not manageable in terms of the final result, an expander instead of an implant was used.

### 2.3. ICG Angiography Administration Protocol

The IC-FlowTM system (developed by Seda s.p.a., Milan, Italy) was utilized to perform the intraoperative laser-assisted green indocyanine fluoroangiography [30]. Following completion of mastectomy and prior to breast implant insertion, a single dose (0.2 mg/kg) of VERDYE^®^ vital dye (5 mg/mL concentration sourced from a 25 mg vial of injectable powder diluted with sterile water as prescribed in the drug information leaflet by Diagnostic Green Ltd., Westmeath, Ireland) was administrated via a standard peripheral venous catheter, followed by 10 cc of a saline solution flush. Two minutes post-administration, infrared scanning of skin flaps was performed using the IC-FlowTMImagingSystem. In order to avoid interference of ambient light, all operating room lights were dimmed.

**Figure 1:**
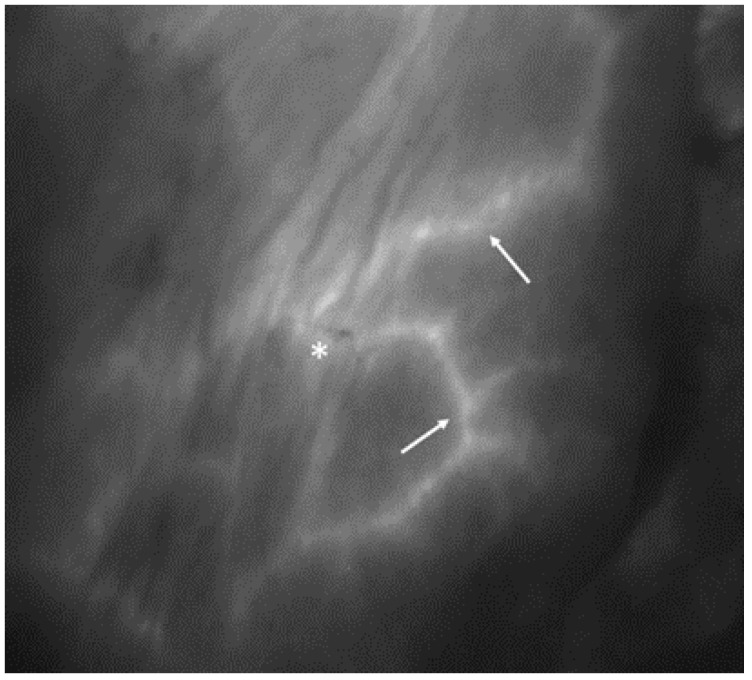
ICG angiography following mastectomy. The asterisk indicates the location of the nipple, and the white arrows highlight the small terminal branches of subcutaneous capillary vessels, exhibiting contrast enhancement and adequate perfusion.

**Figure 2:**
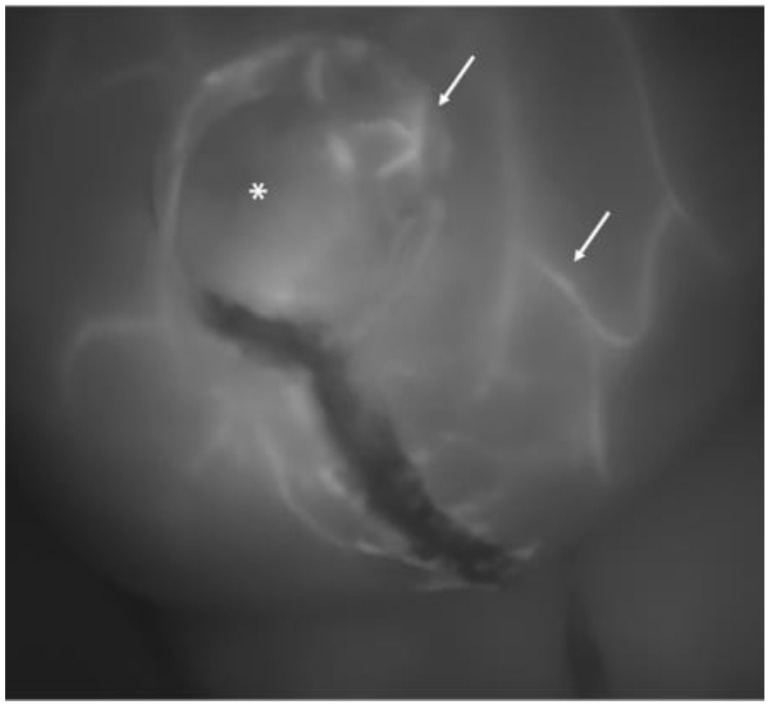
Another image of a breast at the completion of the procedure: the asterisk indicates a skin perforator vessel from which several small tangential vessels branch out (white arrows). This pattern is characteristic of a well-perfused skin flap.

When the contrast permeated the skin layer through the capillary system, the grayscaled screen showed the radially extending pattern of vascularization from the perforator vessels (Figure 1 and Figure 2).

**Figure 3:**
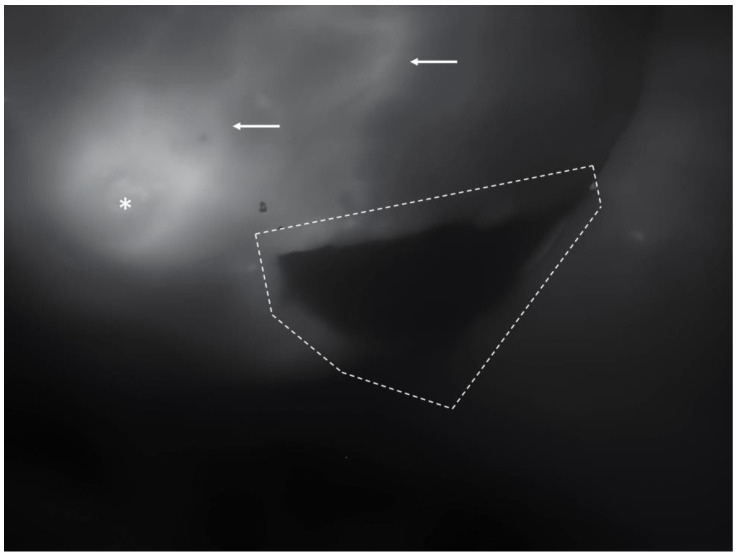
An example of a non-perfused skin area: inferiorly to the nipple (asterisk), a black area is demarked by dotted lines. In contrast, the surrounding skin (white arrows) displays evident perfusion.

Any skin areas indicating inadequate perfusion that appeared black on the screen were promptly excised (Figure 3). The handpiece of the angiography device features controls to adjust digital image brightness and contrast, facilitating visualization of contrast enhancement during examination. In addition, the distance between the skin surface and the optics on the handpiece was adjusted—as in a normal camera, where the focal distance can be varied to reach the focus point—to provide a more precise observation of capillary refill.

### 2.4. Statistical Analysis

The statistical analysis focused specifically on complications associated with vascular impairment. To compare the complication rates between arm A and arm B, Fisher’s exact test was utilized. Additionally, the association between reported risk factors from the literature and complications was assessed using the chi-square test, with Yates correction applied for the sample size. A univariate analysis was performed to identify any correlation between mastectomy skin flap necrosis and the aforementioned risk factors. Finally, the effect of risk factors on the complications variable was calculated using a multivariate regression analysis. The statistical analysis was performed using the R Project for statistical computing.

## 3. Results

During the study period, a total of 50 patients were included. All patients underwent mastectomy followed by an implant reconstruction. Biometric data for all the patients are shown in Table 1. There were some differences observed between the two groups in terms of average age and body mass index, without, however, causing a problem for the homogeneity of the groups according to our statistical analysis.

The prevalence of risk factors in both groups was also considered. The risk factors considered are listed in Table 2.

In Group A, there were 15 nipple-sparing mastectomies (60%), 6 skin-sparing mastectomies (24%), and 4 skin-reducing mastectomies (16%) performed. In 10 cases (30%), surgical incisions were made in the central quadrant with NAC removal, while in 15 patients (60%), they were made in the superior-lateral quadrant using an S-shaped incision. Eleven patients (44%) underwent reconstruction with implants (Mentor^®^ CPG 312, CPG 321, or CPG 322). In the remaining 14 patients, a tissue expander (Mentor^®^ expanders CPX 275 cc, 350 cc, 450 cc, or 550 cc) was preferred for a two-step reconstruction process. Among direct-to-implant patients, 8 (72.7%) received implants larger than 300 cc, with an average implant volume of 392 cc (ranging from 180 cc to 755 cc). Only one patient (7%) had a tissue expander filled with more than 400 cc of saline solution, with theaverage filling volume of the expanders being 110 cc. The placement plane for implants was pre-pectoral in 6 cases (24%) and sub-pectoral in 19 (76%). The average duration of surgery was 225.37 min, and the average hospital stay was 2.36 days.

Group B comprised 14 patients undergoing nipple-sparing mastectomies (56%), 8 skin-sparing mastectomies (32%), and 3 skin-reducing mastectomies (12%). Among these, in 10 cases (40%), the surgical incision was placed in the central quadrant with NAC removal, in 14 cases (56%) in the superolateral quadrants, and in 1 case (4%) in the inframammary fold. Reconstruction was performed in 22 patients (88%) using a direct-to-implant technique (Mentor^®^implants CPG 312, CPG 321, or CPG 322 (Mentor Medical Systems B.V., Zernikedreef 2, 2333CL Leiden, The Netherlands)), while the remaining 3 patients (12%) underwent reconstruction with a tissue expander (Mentor^®^ expander CPX 350 cc, 450 cc, or 550 cc). In 17 cases (77%), the implant exceeded 300 cc, with an average volume of 396 cc (ranging from 180 cc to 570 cc). The tissue expanders were never filled beyond 300 cc, with an average volume of 200 cc. Implant placement was prepectoral in 4 patients (16%) and subpectoral in 21 patients (84%). The average surgical duration was 215.54 min, with an average hospital stay of 2.56 days.

The overall complication rates are presented in Table 3. In Group A, we observed an overall complication rate of 28% (*n* = 7). Among these complications, 16% (*n* = 4) were classified as major, necessitating revision surgery, while 12% (*n* = 3) were considered minor and did not require further intervention. Specifically, the complications included one early seroma, two cases of superficial MSFN, and two instances of severe MSFN with prosthetic exposure. The rate of revision surgery was 16%.

Group B recorded a complication rate of 36% (*n* = 9), with 24% (*n* = 6) classified as major and 12% (*n* = 3) as minor. Specifically, the complications included one early seroma, two cases of superficial mastectomy skin flap necrosis (MSFN), and six instances of full-thickness MSFN with prosthetic exposure requiring revision surgery. The reoperation rate in Group B was reported as 24%.

When considering the type of implant used in patients who developed severe MSFN requiring surgical revision, it was found that in group A, a prosthesis was placed in three patients, while an expander was placed in one patient. In contrast, all six patients in group B received a prosthesis. When exclusively considering the placed prostheses, the complication rate stoodat 27% in both groups.

Statistical analysis was focused on complications related to blood supply deficiency and on reoperation rate. Despite the lower complication rate reported in Group A, no significant differences were found in mastectomy skin flap necrosis rate and reoperation rate between the two groups.

Risk factors were investigated in all patients who experienced complications. The correlation between severe skin flap necrosis and the recorded risk factors is detailed in Table 4. The only factor demonstrating a significant association with MSFN was previous surgery (*p* = 0.032).

## 4. Discussion

Skin flap necrosis and other complications related to impaired blood perfusion can be devastating events. Beyond prolonged hospitalization, delayed wound healing, and compromised outcomes of reconstructive procedures, there are additional concerns. Mastectomy skin flap necrosis can also delay the timely administration of adjuvant therapy and have a psychological impact on the patient [17]. Typically, the perfusion of the skin flap is assessed by the surgeon through clinical evaluation following mastectomy. This evaluation involves assessing skin color, temperature, capillary refill, and dermal bleeding from incision edges. However, these criteria rely on subjective interpretation.

Many authors have reported a significantly high rate of mastectomy skin flap necrosis (MSFN) following clinical assessment of skin flap perfusion. The incidence of skin flap necrosis following mastectomy can be as high as 40% in the literature [14].

To enhance the accuracy of skin flap assessment, many authors have suggested ICG angiography as a reliable and more objective tool, assisting surgeons in reducing the occurrence of skin flap necrosis [27,31]. The literature supports a reduction in the risk of complications and reconstruction failure through a comprehensive meta-analysis and review of the latest studies on indocyanine green angiography [32,33]

ICG angiography has demonstrated high accuracy in both the literature and our study [28]. Specifically, when considering only severe necrosis requiring revision surgery, we found a specificity of 100%, a sensitivity of 75%, and a negative predictive value of 95%.

Only three patients experienced ischemic complications, even though ICG did not detect any perfusion deficits. Two patients developed mild necrosis, which was managed conservatively and resolved with medication, while one patient developed full-thickness necrosis necessitating reoperation.

In the first case, the patient had a large breast size (cup size > 5) and a BMI > 25 kg m^2^. Despite ICG angiography indicating good flap viability, we opted to implant a high-volume prosthesis (Mentor CPG321, 755 cc) to achieve symmetrical reconstruction, in line with the patient’s preference to maintain her original breast volume and avoid any reduction procedures. The patient developed an ischemic complication but wound healing was successfully completed in 87 days, with no need for revision surgery.

In the second case, the patient had a small breast volume and did not present any risk factors. ICG angiography confirmed good perfusion of the mastectomy flaps, and as the patient requested augmentation, we opted for a direct-to-implant (DTI) reconstruction using a 420 cc Mentor CPG 322 implant. Three days post-surgery, mild necrosis occurred. No reintervention was necessary, and the complication was resolved with conservative treatment within 32 days.

In the third case, the patient had risk factors including previous breast surgery and smoking habits, but no flap perfusion impairment was observed during intraoperative ICG angiography. The patient underwent a direct-to-implant (DTI) reconstruction using a high-volume implant (515 cc CPG 322 Mentor). However, three days post-surgery, the patient developed severe mastectomy skin flap necrosis (MSFN) necessitating surgical revision and implant removal. During the reoperation, necrotic tissues were debrided, and the implant was replaced with a 315 cc implant. Wound healing was completed within two weeks without any further complications.

Despite the small size of our sample, which made it challenging to identify a statistically significant association between prosthesis volume and ischemic complications (*p* = 0.06), we still believe that the implant size may have played a role in causing superficial necrosis. The association between implants and mastectomy skin flap necrosis is in fact well-documented in the literature [19,20]. The weight of the implant, combined with the risk factors present in the first and third patients, likely had a detrimental effect on skin vascularization. In line with this theory, we observed mild necrosis primarily in the lower pole, where the skin stretching due to the weight of the implant was most pronounced.

Consistent with the literature, in Group B, where skin flap perfusion was evaluated clinically, we observed an overall complication rate of 36%. When focusing on complications arising from vascular impairment requiring revision surgery, the complication rate decreased to 24%. In Group A, where ICG angiography was utilized to detect perfusion impairment, the overall complication rate decreased to 16%. When considering only full-thickness necrosis necessitating a return to the operating room, the complication rate decreased to 8%. Several studies have documented a reduction in full-thickness necrosis with the use of ICG angiography compared to clinical evaluation alone [34,35,36]. Although we did not establish a significant decrease due to the limited sample size, the decrease in skin flap necrosis aligns with findings in the literature.

These data suggest the effectiveness of intraoperative assessment of skin flaps using ICG angiography compared to the outcomes observed in group B, where only a clinical evaluation was conducted to assess potential blood deficiency.

ICG angiography actively guides the surgeon in the reconstructive plan. Integrating ICG angiography into the surgical decision-making process not only enhances surgical precision but also allows for a more tailored approach to reconstruction. By identifying areas of impaired vessel flow and resecting them, surgeons can proactively address potential ischemic complications, thereby optimizing patient outcomes and reducing the risk of postoperative complications and any potential delays in healing.

When ICG angiography reveals poor local perfusion, the surgeon can choose between simply resecting the incision edges or opting for both incision edge resection and exploring alternative reconstructive options. In cases where vascular impairment is widespread, the surgeon may opt for a two-stage reconstructive plan. Instead of using an implant, an underfilled expander may be placed to mitigate the risk of skin ischemia, as previously described in the literature [37,38]. These actions effectively prevented the onset of mastectomy skin flap necrosis and the failure of immediate reconstruction.

Confirming the previous statement, group A exhibitedhigher rates of reconstruction with expanders compared to group B (56% vs. 2%, respectively). However, the inclusion of the expansion constituted a limit, depriving this study of homogeneity given that the weight of the expander is variable. When there is uncertainty about poorly vascularized mastectomy flaps, surgeons tend to opt for a more aggressive debridement and adjust the reconstructive plan accordingly. This aspect is also emphasized in the literature as a key advantage of ICGA [39].

While ICG angiography is widely available in many institutions, there remains a lack of consensus on its protocol and relative effectiveness. The infrared angiography device utilized in our study presents its own challenges, notably requiring a learning curve to interpret the grayscaled signals. This could introduce variability in the assessment and decision-making process among surgeons, highlighting the need for standardized protocols and training programs to ensure a consistent and accurate interpretation of ICG angiography results. Moreover, the imaging lacks a correlation withnumerical values and does not graphically indicate distinct areas of perfusion on the screen, aside from the indocyanine green contrast. The lack of quantitative data from the imaging may limit its utility in guiding surgical decisions and can lead to an overestimation or underestimation of the vascular impairment. In our cases, the underestimation may be attributed to various factors, including the operating room light, other light sources, and low circulatory pressure, necessitating self-interpretation by the surgeon and potentially leading to differing interpretations among surgeons.

We encountered this issue in three patients who developed ischemic complications despite no detectable perfusion impairment on ICG examination. In such cases, we cannot rule out the possibility of underestimation of the grayscale image.

To address these challenges, systems such as the SPY Intraoperative Perfusion As-sessment System (distributed in North America by LifeCell Corp., Branchburg, NJ, USA; manufactured by Novadaq Technologies Inc., Richmond, BC, Canada) have been proposed to quantify the spread of ICG in ischemic tissues. However, further analysis of their cost-effectiveness is needed [40].

### Limitations

Despite the valuable insights provided by this study, several limitations should be considered. This study’s sample size was relatively small, which may limit the generalizability of the findings and made a stratified analysis impractical. Larger studies involving a more diversified patient population are needed to confirm the observed trends and associations. Moreover, this study did not include a preoperative assessment of patient skin thickness, which could have provided additional insights into tissue characteristics and influenced our surgical planning decisions. This information might have influenced decision-making at the conclusion of mastectomy or even during preoperative planning [41]. Finally, interpretation of ICG angiography results can be subjective and may vary among surgeons. Standardized protocols and training programs are necessary to ensure there is a consistent and accurate interpretation of ICG angiography findings, thereby reducing potential variability in surgical decision-making. Future studies could test the variability among observers.

## 5. Conclusions

This study underscores the importance of intraoperative evaluation in preventing complications such as MSFN. Especially in patients with numerous risk factors, the integration of ICG angiography into the surgical workflow allows for a more tailored approach to reconstruction. Objective assessment of mastectomy flap vascularization quality enables surgeons to confidently perform more aggressive tissue debridement and to feel more confident in changing the reconstructive plan. In future studies, efforts should focus on refining ICG angiography protocols, improving image interpretation techniques, and evaluating the impact of interobserver variability in image interpretation. Standardized protocols and training programs will be essential to ensure the consistent and accurate interpretation of ICG angiography results.

## Figures and Tables

**Table 1:** Biometric characteristics of the study population in this two-arm study.

Feature	Arm A	Arm B	*p*-Value
**Age**	54.53 ± 9.98 (30–73)	53.12 ± 9.18 (37–75)	0.00017
**Weight (kg)**	64.3 ± 13.25 (50–100)	63.4 ± 10.66 (45–84)	0.4
**BMI**	23.68 ± 4.58 (17.9–36.7)	23.05 ± 3.89 (17.6–30)	0.0014
**Cup size**			>0.05
Small (≤250 gr)	8 (32%)	10 (40%)	
Medium (250–450 gr)	10 (40%)	7 (28%)	
Large (≥450 gr)	7 (28%)	8 (32%)	
**Mastectomy volume**	286.5 ± 149.206 (125–750)	382.29 ± 134.39 (150–585)	0.0014

**Table 2:** The distribution of risk factors among ICG (arm A) and clinically assessed patients (arm B).

Risk Factor	Arm A	Arm B	*p*-Value
**Age > 50**	76% (19)	64% (16)	>0.05
**Smoking**	36% (9)	28% (7)	>0.05
**Diabetes**	8% (2)	4% (1)	>0.05
**BMI > 25**	16% (4)	16% (4)	>0.05
**Cup size > medium**	68% (17)	72% (18)	>0.05
**Neoadjuvant RT**	28% (7)	8% (2)	>0.05
**Previous surgery** (same breast)	36% (9)	16% (4)	>0.05
**Type of mastectomy**			>0.05
Nipple sparing	60% (15)	56% (14)	
Skin sparing	24% (6)	32% (8)	
Skin reducing	16% (4)	12% (3)	
**Incision**			>0.05
Central	40% (10)	40% (10)	
Upper-lateral	60% (15)	56% (14)	
Inframammary fold	-	4% (1)	
**Type of reconstruction**			
**Prothesis** **Direct-to-implant**	44% (11)	88% (22)	0.0023
Volume > 400 cc	72% (8)	77% (17)	
Volume < 400 cc	28% (3)	23% (5)	
**Expander**	56% (14)	2% (3)	0.0023
Volume > 400 cc	7% (1)	-	
Volume < 400 cc	93% (13)	100% (3)	

**Table 3:** Complication and revision surgery rates between patients treated with ICG (Arm A) and those evaluated clinically (Arm B).

	Total	Arm A	Arm B	*p*-Value
**Complications**	16	28% (7)	36% (9)	0.61
**Skin flap necrosis**	12	16% (4)	32% (8)	0.32
Full thickness	8	8% (2)	24% (6)	0.31
Superficial	4	8% (2)	8% (2)	1
**Early seroma**	2	4% (1)	4% (1)	1
**Hematoma**	2	8% (2)	-	0.50
**Revision surgery rates**	10	16% (4)	24% (6)	0.32
Due to skin necrosis	8	8% (2)	24% (6)	0.31
Due to other complications	2	8% (2)	-	0.50

**Table 4:** Incidence of risk factors in the group of patients who developed full-thickness necrosis.

Risk Factor	Full-Thickness Necrosis % (*n*)	*p*-Value
Age > 50	50% (4)	0.42
Smoking	25% (2)	0.91
Diabetes	12.5% (1)	0.13
BMI > 25	50% (4)	0.70
Cup size > medium	87.5% (7)	0.85
Radiotherapy	12.5% (1)	0.06
Previous surgery	37.5% (3)	0.03
NSSM	62.5% (5)	0.36
Volume> 400 cc	75% (6)	0.06

## Data Availability

The original contributions presented in the study are included in the article, further inquiries can be directed to the corresponding author/s.
